# Metabolic engineering of *Acinetobacter baylyi* ADP1 for removal of *Clostridium butyricum* growth inhibitors produced from lignocellulosic hydrolysates

**DOI:** 10.1186/s13068-015-0389-6

**Published:** 2015-12-01

**Authors:** Matti S. Kannisto, Rahul K. Mangayil, Ankita Shrivastava-Bhattacharya, Brett I. Pletschke, Matti T. Karp, Ville P. Santala

**Affiliations:** Department of Chemistry and Bioengineering, Tampere University of Technology, Korkeakoulunkatu 8, Tampere, Finland; Department of Biochemistry and Microbiology, Enzyme Synergy Programme, Rhodes University, Grahamstown, 6140 South Africa

**Keywords:** *Acinetobacter baylyi*, *Clostridium butyricum*, Metabolic engineering, Rice straw hydrolysate, Biodetoxification, Biohydrogen

## Abstract

**Background:**

Pretreatment of lignocellulosic biomass can produce inhibitory compounds that are harmful for microorganisms used in the production of biofuels and other chemicals from lignocellulosic sugars. Selective inhibitor removal can be achieved with biodetoxification where microorganisms catabolize the inhibitors without consuming the sugars. We engineered the strictly aerobic *Acinetobacter baylyi* ADP1 for detoxification of lignocellulosic hydrolysates by removing the gene for glucose dehydrogenase, *gcd*, which catalyzes the first step in its glucose catabolism.

**Results:**

The engineered *A. baylyi* ADP1 strain was shown to be incapable of consuming the main sugar components of lignocellulosic hydrolysates, i.e., glucose, xylose, and arabinose, but rapidly utilized acetate and formate. Formate was consumed during growth on acetate and by stationary phase cells, and this was enhanced in the presence of a common aromatic inhibitor of lignocellulosic hydrolysates, 4-hydroxybenzoate. The engineered strain tolerated glucose well up to 70 g/l, and the consumption of glucose, xylose, or arabinose was not observed in prolonged cultivations. The engineered strain was applied in removal of oxygen, a gaseous inhibitor of anaerobic fermentations. Co-cultivation with the *A. baylyi* ADP1 *gcd* knockout strain under initially aerobic conditions allowed the strictly anaerobic *Clostridium butyricum* to grow and produce hydrogen (H_2_) from sugars of the enzymatic rice straw hydrolysate.

**Conclusions:**

We demonstrated that the model organism of bacterial genetics and metabolism, *A. baylyi* ADP1, could be engineered to be an efficient biodetoxification strain of lignocellulosic hydrolysates. Only one gene knockout was required to completely eliminate sugar consumption and the strain could be used in production of anaerobic conditions for the strictly anaerobic hydrogen producer, *C. butyricum*. Because of these encouraging results, we believe that *A. baylyi* ADP1 is a promising candidate for the detoxification of lignocellulosic hydrolysates for bioprocesses.

## Background

Lignocellulosic biomass is widely considered as one of the most promising substrates for biological production of fuel molecules such as H_2_, but due to its recalcitrant nature, the raw material has to be subjected to harsh pretreatment steps [1]. Pretreatment may release molecules such as organic acids, furans, and aromatic compounds, which are often inhibitory to growth and product formation in bioprocesses [2]. Inhibitor concentrations vary greatly depending on the lignocellulosic biomass and pretreatment conditions, and in some hydrolysates acetate is found in concentrations as high as 10 g/l [3]. At low concentrations some inhibitors might have a positive effect on a bioprocess, e.g., 3.3 g/l of the undissociated form of acetate has a positive effect on ethanol yield of *S. cerevisiae* [4]. However, at elevated concentrations (5 g/l of the undissociated form) acetate becomes detrimental to its growth [4], and this effect occurs even at lower concentrations of acetate when xylose is used as a carbon source instead of glucose [5]. Acetate has also been shown to increase the toxicity of furfural on *Escherichia coli* [6] and *Saccharomyces cerevisiae* [7]. Thus, even though acetate itself is not a strong inhibitor, its removal from lignocellulosic hydrolysates could decrease the overall toxicity of furfural. The removed acetate could serve as a substrate for lipid synthesis since it is usually catabolized via acetyl-CoA intermediate which is also a starting point for synthesis of lipids. For example, *Acinetobacter baylyi* ATCC 17976 accumulates more than 10 % of the dry weight of wax esters when grown on acetate under nitrogen limitation [8].

Physicochemical and microbiological methods for the detoxification of lignocellulosic hydrolysates have been developed. The former methods are often more rapid but they can decrease the sugar yield, and the toxin removal may be incomplete, while the latter methods require less energy and produce less waste water [9]. Some microorganisms are able to utilize the inhibitors as a carbon source, which can be used for the production of biochemicals. For example, furanic compounds can be catabolized mainly by aerobic Gram-negative bacteria, like *Cupriavidus basilensis* HMF14 [10] and also by certain fungi and *Acinetobacter* strains [11]. Thus biodetoxification would satisfy several requirements that have been considered to be important for making lignocellulosic ethanol more cost-effective [12]. Biodetoxification of lignocellulosic hydrolysates has been attempted with microorganisms engineered to perform this function [13, 14] or by isolating microorganisms that are naturally capable of consuming these inhibitors without decreasing sugar yield [15]. Model organisms have the advantage that vast amounts of knowledge have accumulated about their biology and they often can be used to produce industrially relevant biochemicals. However, it has proved to be difficult to completely eliminate sugar consumption by model organisms such as *E. coli* [13] and *S. cerevisiae* [14].

In addition to the inhibitors derived from the pretreatment of lignocellulosic biomass, other growth-inhibiting molecules might be present in the cultivation media that have to be removed prior to fermentation. For example, biological H_2_ production with anaerobic bacteria such as of the genus *Clostridia* cannot be carried out before oxygen has been removed. Traditionally, this has been achieved by physical treatments such as boiling and flushing with nitrogen (N_2_) or by addition of chemicals, such as cysteine-HCl and sodium sulfide, but oxygen can also be removed by cultivating aerobic bacteria. For example, Tran et al. [16] have used *Bacillus subtilis* in saccharification of starch and oxygen removal which enhanced biohydrogen production by *Clostridium butylicum* and eliminated the need for expensive reducing agents and N_2_ flushing [16].

*Acinetobacter baylyi* ADP1 has become a model bacterium for studies of genetics and metabolism due to its wide substrate spectrum and natural tendency to take up and incorporate foreign DNA into its genome [17]. These studies have led to an accumulation of large amounts of knowledge about the biology of this bacterium [18–20], and especially aromatic compound catabolism [21] and natural transformation [22] have been studied intensively. *A. baylyi* ADP1 has also been engineered for the production of valuable biochemicals, like cyanophycin [23], wax esters [24], and triacylglycerols [25]. As *A. baylyi* strains are known for their inability to grow on most sugars as a sole carbon source, we considered that *A. baylyi* ADP1 would be an ideal organism for the detoxification of lignocellulosic hydrolysates. The only sugar that *A. baylyi* ADP1 can use as a sole carbon source is glucose, and it can only catabolize glucose if it is first converted to gluconate by glucose dehydrogenase [18] which makes metabolism of *A. baylyi* ADP1 easy to modify for the biodetoxification of lignocellulosic hydrolysates.

We disabled *A. baylyi* ADP1's sugar catabolism by removing the gene for glucose dehydrogenase, *gcd*, from its genome. The strain was tested for its ability to remove acetate and other common organic acids encountered in lignocellulosic hydrolysates from a growth medium without affecting the concentrations of sugars. We also determined the effect of a model phenolic inhibitor of lignocellulosic hydrolysates, 4-hydroxybenzoate, on the consumption of acetate and formate. Performance of the strain was assessed at high glucose concentrations and in simulated lignocellulosic hydrolysates containing glucose, xylose, and arabinose at elevated concentrations. Finally, the strain was also used in the removal of oxygen for H_2_ production by *C. butyricum*.

## Results

### Characterization of the strains

**Fig. 1:**
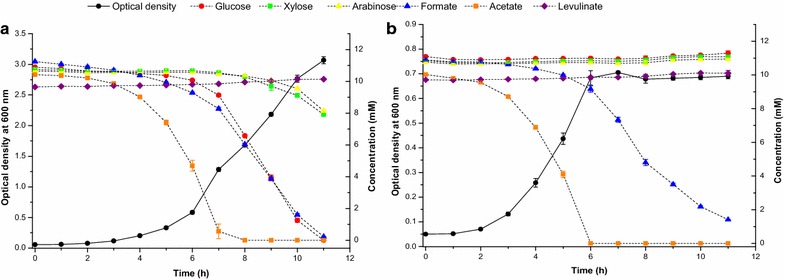
Growth and consumption of d-glucose, d-xylose, l-arabinose, acetate, formate, and levulinate of wild-type *A. baylyi* ADP1 (**a**) and *gcd* knockout strain (**b**). Cultivations were carried out in triplicate and the results are shown as averages with *error bars* representing standard deviations

In order to characterize the growth of *A. baylyi* ADP1 and *gcd* knockout strain on a mixture of sugars and organic acids commonly found in most lignocellulosic hydrolysates, we cultivated the cells on approximately 10 mM of glucose, xylose, arabinose, formate, acetate, and levulinate (Fig. 1). The wild-type cells consumed glucose, formate, and acetate simultaneously. Acetate was consumed the most rapidly, and the growth rate was the highest when it was available as a carbon source, but it did not seem to repress catabolism of glucose or formate to great extent. Xylose and arabinose were oxidized less preferably than glucose. The *gcd* knockout strain grew slightly faster (µ = 0.58 ± 0.01 h^−1^) than the wild-type strain (µ = 0.50 ± 0.02 h^−1^) during the beginning (2–6 h) of the cultivation, without consuming any of the sugars, but its growth ceased when acetate was depleted. However, formate consumption continued after this point and it seems that it cannot be used as a sole carbon source for growth but can be still be consumed by stationary phase cells. Acetate consumption was slightly faster than with wild-type cells but formate was consumed less rapidly. Neither strain was able to catabolize levulinate and this molecule was therefore excluded from subsequent experiments.

**Fig. 2:**
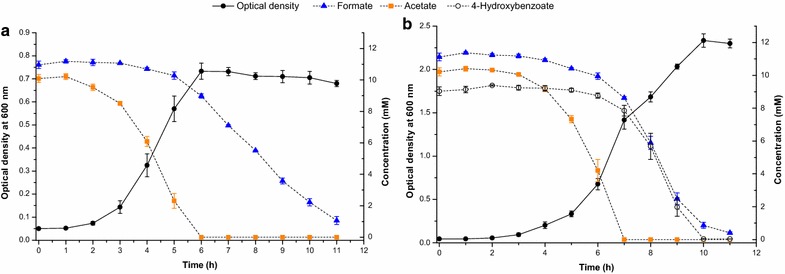
*A. baylyi* ADP1 *gcd* knockout strain’s growth and consumption of acetate and formate in the absence (**a**) or presence (**b**) of 4-hydroxybenzoate. The data are averages of triplicate cultivations and *error bars* show standard deviations

Since *A. baylyi* ADP1 is able to catabolize many of aromatic inhibitors found in lignocellulosic hydrolysates [17], we tested the ability of the *gcd* knockout strain to consume 4-hydroxybenzoate, an aromatic inhibitor common in some hydrolysates [26], in the presence of acetate and formate (Fig. 2). The presence of acetate prevented consumption of 4-hydroxybenzoate. Formate, on the other hand, did not prevent 4-hydroxybenzoate consumption and the presence of 4-hydroxybenzoate allowed faster removal of formate. The presence of 4-hydroxybenzoate, however, decreased acetate consumption rate slightly.

### Growth at elevated sugar concentrations

**Fig. 3:**
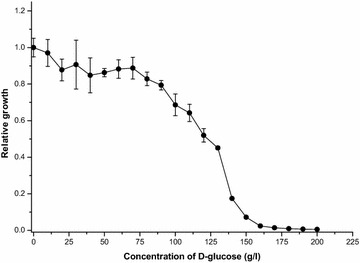
*A. baylyi* ADP1 *gcd* knockout strain’s relative growth on 4 g/l acetate at different d-glucose concentrations after 24 h of cultivation. Relative growth values were calculated by dividing the optical densities by an average optical density value at 0 g/l d-glucose. The data are averages from triplicate cultivations with *error bars* representing standard deviations

As glucose concentration in lignocellulosic hydrolysates varies greatly depending on the source and pretreatment of the biomass, we tested the ability of the *gcd* knockout strain to grow on 4 g/l acetate at glucose concentrations from 0 to 200 g/l (Fig. 3). The presence of the sugar in the medium, at concentrations below 70 g/l, inhibited growth only to a very modest degree. Increasing the glucose concentration above this level began to affect growth to a much greater extent, although the cells grew even at glucose concentration of 150 g/l. Thus it seems that high concentration of glucose, usually the most abundant sugar in lignocellulosic hydrolysates, should not become detrimental to the growth of *A. baylyi* ADP1 *gcd* knockout mutant.

**Fig. 4:**
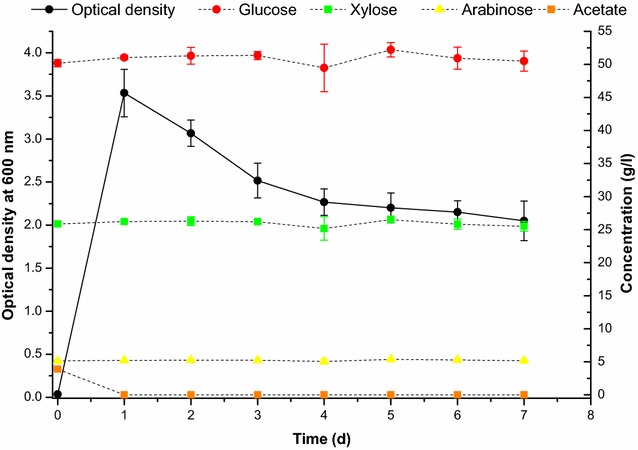
*A. baylyi* ADP1 *gcd* knockout strain’s growth and concentrations of d-glucose, d-xylose, l-arabinose, and acetate during a prolonged cultivation in a simulated lignocellulosic hydrolysate. The cultivations were carried out in triplicate and averaged results are shown with standard deviations

**Fig. 5:**
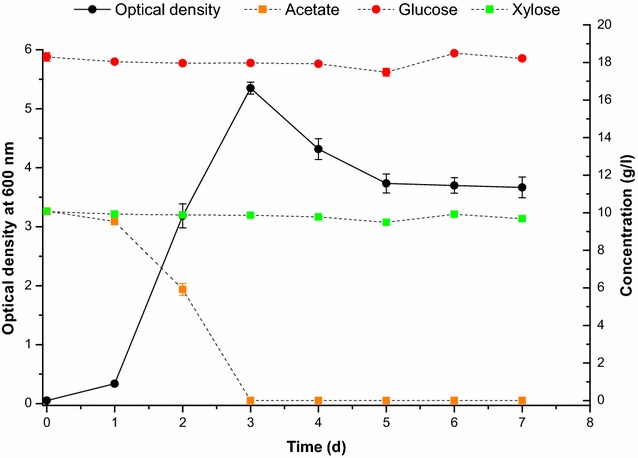
Growth and consumption of d-glucose, d-xylose, and acetate of the *A. baylyi* ADP1 *gcd* knockout strain during a prolonged cultivation in a simulated lignocellulosic hydrolysate with a high concentration of acetate. The data shown are averages from triplicate cultivations with *error bars* showing standard deviations

Performance of the *A. baylyi* ADP1 *gcd* knockout strain in the removal of acetate from a medium containing high concentrations of sugars was tested by cultivating the strain for a prolonged period of time, in a medium simulating the sugar fractions of a lignocellulosic hydrolysate (Fig. 4). The strain did not consume any of the sugars, glucose (50 g/l), xylose (25 g/l), or arabinose (5 g/l), after 7 days of cultivation but depleted the medium of acetate (4 g/l) after 1 day of cultivation. The knockout strain was cultivated at higher acetate concentration (10 g/l) in the presence of glucose (20 g/l) and xylose (10 g/l) in order to show that consumption of these sugars does not occur when cells have grown to higher densities (Fig. 5).

### Oxygen removal for H_2_ production by *C. butyricum*

The ability of *A. baylyi* ADP1 *gcd* knockout strain to remove oxygen from a sealed container without affecting sugar concentrations was tested by cultivating the knockout strain and *C. butyricum*, either alone, or in co-cultivations using enzymatic rice straw hydrolysate as a substrate. Rice straw hydrolysate contained mainly cellobiose, glucose, and xylose as sugars—and citrate (from the buffer components used for the cellulolytic enzyme hydrolysis reaction). The lignocellulosic hydrolysate was added to a minimal medium which contained 0.3 g/l yeast extract. It was intended that *A. baylyi* ADP1 *gcd* knockout strain would grow in the medium using citrate as a carbon source and deplete the medium of oxygen. After this, the strictly anaerobic *C. butyricum* could start to grow and produce H_2_.

After 24 h of cultivation in sealed vessels it could be seen that *C. butyricum* had not increased in optical density at 600 nm, consumed or produced any of the main metabolites or produced H_2_ under aerobic conditions, indicating that no growth had occurred (Table 1). The *A. baylyi* ADP1 *gcd* knockout strain had grown to a modest degree but had not produced H_2_. The sugar concentrations had not changed significantly in the cultivation of the *A. baylyi* ADP1 *gcd* knockout strain but a small decrease in concentrations of acetate and citrate had occurred. The presence of the knockout strain in the aerobic cultivation vessel allowed growth of *C. butyricum*. When compared to cultivation of *C. butyricum* under anaerobic conditions, the co-cultivation produced H_2_ in similar amounts but consumed more glucose and xylose while producing more acetate and butyrate. Cellobiose consumption on the other hand was slightly higher in anaerobic cultivation of *C. butyricum* than in the co-cultivation. Of the total mass of the sugars, *C. butyricum* (under initially anaerobic conditions) consumed 44.2 ± 0.6 % of the sugars while 49.0 ± 0.4 % of the sugars was consumed in the co-cultivation.

**Table 1:** Optical densities at 600 nm, H_2_ production, and concentrations of main metabolites at beginning and end of cultivations of *C. butyricum* and *A. baylyi* ADP1 *gcd* knockout strain

	*C. butyricum* (anaerobic)		*C. butyricum*		*A. baylyi*		*C. butyricum* and *A. baylyi*	
	0 h	24 h	0 h	24 h	0 h	24 h	0 h	24 h
OD_600_	0.5 ± 0.0	>3.0	0.5 ± 0.0	0.4 ± 0.0	0.2 ± 0.0	1.1 ± 0.0	0.7 ± 0.1	>3.0
H_2_ (mM)	‒	93.6 ± 3.6	‒	0.0 ± 0.0	‒	0.0 ± 0.0	‒	92.0 ± 1.5
c_Glucose_ (mM)	45.8 ± 1.1	15.5 ± 0.6	45.4 ± 0.5	46.1 ± 1.0	45.5 ± 0.7	45.0 ± 0.4	45.0 ± 0.4	5.5 ± 0.1
c_Cellobiose_ (mM)	34.5 ± 0.9	17.6 ± 0.1	34.6 ± 0.4	34.7 ± 0.7	34.7 ± 0.5	34.6 ± 0.3	34.2 ± 0.2	19.4 ± 0.3
c_Xylose_ (mM)	40.5 ± 1.0	38.3 ± 0.6	40.5 ± 0.4	40.9 ± 0.6	40.2 ± 0.6	39.2 ± 0.4	40.0 ± 0.3	36.9 ± 0.6
c_Acetate_ (mM)	1.9 ± 0.1	27.5 ± 0.1	1.8 ± 0.1	2.2 ± 0.3	1.2 ± 0.0	0.2 ± 0.2	1.8 ± 0.1	28.6 ± 0.6
c_Butyrate_ (mM)	0.7 ± 0.0	31.9 ± 0.3	0.7 ± 0.0	0.9 ± 0.3	0.0 ± 0.0	0.0 ± 0.0	0.7 ± 0.1	34.9 ± 0.7
c_Citrate_ (mM)	34.4 ± 0.8	29.9 ± 0.4	34.9 ± 0.4	35.3 ± 0.7	34.1 ± 0.5	31.7 ± 0.3	33.8 ± 0.3	29.5 ± 0.6

Results shown are averages with standard deviations from triplicate cultivations

## Discussion

*A. baylyi* ADP1 does not have a complete Embden–Meyerhof–Parnas glycolytic pathway and catabolizes glucose only via a modified Entner–Doudoroff pathway [27]. Extracellular oxidation of glucose by glucose dehydrogenase is the first step in this pathway which makes it possible to completely eliminate glucose catabolism by knocking out only one gene, *gcd*. *A. baylyi* is unable to grow on xylose and arabinose as sole carbon sources [28], but they are oxidized by glucose dehydrogenase to sugar lactones, which hydrolyze spontaneously to xylonate and arabinonate at appropriate pH values [29]. Removal of *gcd* from *A. baylyi* ADP1’s genome rendered it unable to oxidize glucose, xylose, or arabinose, while it readily consumed acetate and formate. Levulinate was not consumed as has been shown by Vaneechoutte et al. [30]. Formate, which was consumed slower than acetate, could not be used as a single carbon source for growth but was consumed by stationary phase cells. The removal of formate from the medium could be improved by addition of a common aromatic inhibitor of lignocellulosic hydrolysates, 4-hydroxybenzoate. Consumption of this aromatic inhibitor has been shown to be repressed by acetate with *A. baylyi* ADP1 [21] but the presence of formate did not cause this kind of catabolite repression. The presence of 4-hydroxybenzoate inhibited the consumption of acetate. Thus, in addition to being a known inhibitor of microorganisms utilizing lignocellulosic sugars [26], 4-hydroxybenzoate may also act as an inhibitor in the biodetoxification of lignocellulosic hydrolysates. Acetate also represses catabolism of many other aromatic compounds [31], but as this phenomenon is well understood it should be feasible to remove genes responsible for this repression, such as *crc* which codes for catabolite repression control protein Crc [32], from the genome of *A. baylyi* ADP1, if advantageous for bioprocesses.

Removal of acetate with *E. coli* requires multiple gene knockouts and decreased sugar concentrations in the mixture of glucose, xylose, and acetate [13], a drawback which may also occur with detoxification of lignocellulose with chemical methods [2]. *Issatchenkia occidentalis* CCTCC M 206097, although capable of removing several inhibitors from lignocellulosic hydrolysates, decreased glucose and xylose concentrations to varying degrees [33]. This problem was not encountered in our experiments with *A. baylyi* ADP1 engineered for the detoxification of lignocellulosic hydrolysates. Sugar consumption was not observed (even at high sugar concentrations) and the strain performed well at an elevated acetate concentration. The knockout strain was able to completely remove acetate when it was present at 10 g/l. At this concentration, in the presence of furfural, acetate decreases growth and ethanol yield of *S. cerevisiae* [7]. Thus, we have shown that the *gcd* knockout mutant of *A. baylyi* ADP1 is able to selectively remove acetate at a concentration that is significant with respect to utilization of lignocellulosic biomass. The *A. baylyi* ADP1 *gcd* knockout strain is similar to *Cupriavidus basilensis* HMF14 [15] with respect to its ability to remove lignocellulosic inhibitors without decreasing sugar concentrations. Although *C. basilensis* HMF14 is able to remove a broader range of inhibitors, *A. baylyi* ADP1 is easier to modify genetically and has been engineered to produce several industrially relevant biomolecules [23–25]. While *A. baylyi* ADP1 is capable of catabolizing many aromatic inhibitors, such as 4-hydroxybenzoate, vanillate, ferulate, protocatechuate, and benzoate [34], it is unable of growing on furfural or 5-(hydroxymethyl)furfural as sole carbon sources (data not shown). The catabolic abilities of *A. baylyi* ADP1 could be expanded to include inhibitors like furfural and 5-(hydroxymethyl)furfural similarly as has been performed with *Pseudomonas putida* S12 [35].

In addition to removing inhibitors produced in the pretreatment of lignocellulosic biomass, the *A. baylyi* ADP1 *gcd* knockout strain can be used in removing other inhibitors, such as oxygen in this study. Oxygen removal from growth medium for anaerobic fermentations can be done by cultivating aerobic bacteria [16]. We have shown here that this can be accomplished in co-cultivations of *A. baylyi* ADP1’s *gcd* knockout strain and *C. butyricum*. The presence of the detoxification strain made oxygen removal with traditional methods, such as flushing with nitrogen, boiling, and reducing agents, unnecessary. Furthermore, since the oxygen removal was carried out in co-cultivation there was no need for an additional process step. The amount of H_2_ produced did not differ from the cultivation of *C. butyricum* under initially anaerobic conditions, but slightly more sugars were consumed in the co-cultivation. The reason for increased sugar consumption in the co-cultivation could not be determined from these results, but could be due to the consumption of yeast extract by the *A. baylyi* ADP1 *gcd* knockout strain in the oxygen removal phase. We believe that the *A. baylyi* ADP1 *gcd* knockout strain could be used this way in making existing industrial bioprocesses more economically feasible as it can be engineered to selectively consume carbon sources, or other molecules like oxygen, that are harmful to the process.

## Conclusions

We have shown here that the *A. baylyi* ADP1 *gcd* knockout strain is suitable for removing acetate and other abundant inhibitors found in most lignocellulosic hydrolysates without decreasing the concentrations of its sugars. As *A. baylyi* ADP1 has been engineered to produce several valuable biochemicals, it could be used to produce these molecules as a side stream in lignocellulosic biofuel production, while simultaneously making the biofuel production economically more feasible. As *A. baylyi* ADP1 is exceptionally easy to modify genetically and it has been studied to great extent, it can be considered a potential candidate for the biological detoxification of lignocellulosic hydrolysates for bioprocess design.

## Methods

### Construction of the knockout strain

**Fig. 6:**
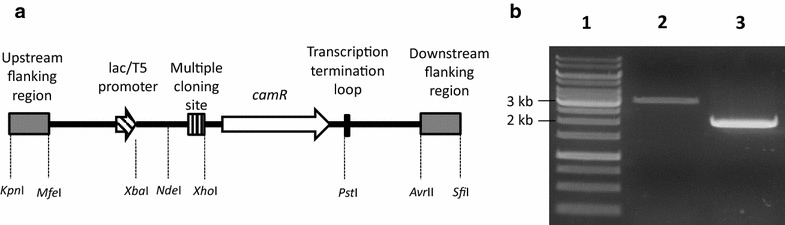
The *gcd* knockout cassette (**a**) and PCR verification of the transformation (**b**). Names of the knockout cassette components are shown above the figure and the sites for restriction enzymes are shown below it. The samples in the *lanes* in B are: *1* GeneRuler™ 1 kb DNA Ladder (Thermo Scientific, Waltham, MA, USA), *2* wild-type *A. baylyi* ADP1, *3* *gcd* knockout mutant of *A. baylyi* ADP1. (**a** adapted from Santala et al. [25])

*Acinetobacter baylyi* ADP1 (DSM 24193) was used in construction of the *gcd* knockout strain and as a control strain in the experiments. The knockout cassette (Fig. 6a) was constructed from the one used previously by Santala et al. [25] using established molecular biotechnology methods [36]. The flanking regions of the cassette were replaced with the flanking regions for knocking out *gcd* (ACIAD2983, NCBI Gene ID: 2878488) by cloning them from the genome of wild-type *A. baylyi* ADP1 with primers having identical annealing regions to those used by de Berardinis et al. [19]. The primers used in construction of the strain are shown in Table 2. The upstream flanking region was cloned with primers U1 and U2 and the downstream flanking region with D1 and D2. Construction of the plasmid was carried out in *Escherichia coli* XL-1 Blue. *A. baylyi* ADP1 was transformed using the method of Metzgar et al. [22] using a PCR product amplified with primers C1 and C2, which annealed to the flanking regions of the cassette. These primers were used also in the verification of the transformants using genomic DNA as a template. A positive transformant gave a product of approximately 2 kb while the genomic DNA of the wild-type cells, which was used as a negative control, gave a product of approximately 3 kb (Fig. 6b).

**Table 2:** Primers used in construction of the *gcd* knockout strain of *A. baylyi* ADP1

Primer	Sequence (5’ → 3’)
U1	GTTTCTTCGGTACCGCTTCTCTCGAATCAACCTAAATG
U2	GTTTCTTCCAATTGGAGACCACCTCGAATAATTTG
D1	GTTTCTTCCCTAGGGCATTGCCGGATACCAAATAATC
D2	GTTTCTTCGGCCCCCGAGGCCCATCGGTGCATAACGCTGTAC
C1	GCTTCTCTCGAATCAACCTAAATG
C2	CATCGGTGCATAACGCTGTAC

### Cultivation of the cells

*A. baylyi* strains were cultivated in an incubator shaker at 30 °C at 300 revolutions per minute (rpm) in 50 ml of a medium with the following composition (g/l): 6.7 K_2_HPO_4_*3H_2_O, 3.4 KH_2_PO_4_, 1.0 NH_4_Cl, 0.3 MgSO_4_, 0.022 CaCl_2_, and 0.004 FeCl_3_. For the initial strain characterization experiment, the medium was supplemented with approximately 10 mM of each d-glucose, d-xylose, l-arabinose, Na-formate, Na-acetate, Na-levulinate. For the experiment carried out to study the effect of an aromatic inhibitor on carboxylic acid consumption, acetate and formate were supplemented at approximately 10 mM with or without approximately 10 mM of 4-hydroxybenzoate. For the glucose tolerance experiment, acetate was used at 4 g/l and glucose at 0 to 200 g/l. For the experiments where the performance of the detoxification strain was evaluated in media simulating lignocellulosic hydrolysate, the media were supplemented with 4 g/l acetate, 5 g/l L-arabinose, 25 g/l D-xylose, and 50 g/l d-glucose, or 10 g/l acetate, 10 g/l D-xylose, and approximately 20 g/l d-glucose. Na-levulinate was prepared from levulinic acid by increasing the pH of the 0.2 M solution to 7 by the addition of NaOH. 4-hydroxybenzoate was prepared similarly but the solution’s pH was increased to 8. In oxygen removal experiments, *A. baylyi* and *C. butyricum* [37] were cultivated at 30 °C at 300 rpm in 25 ml glass tubes in 10 ml of modified version of the medium used by Seppälä et al. [37] (g/l): 1.5 K_2_HPO_4_, 2.0 (NH_4_)_2_SO_4_, 0.2 MgSO_4_*7H_2_O, 0.015 CaCl_2_*2H_2_O, 0.005 FeSO_4_*7H_2_O, 0.3 yeast extract. The medium was supplemented (2 ml/liter) with a trace element solution that contained 1 ml per liter of 25 % HCl and following compounds (mg/l): 70 ZnCl_2_, 100 MnCl_2_*4H_2_O, 60 H_3_BO_3_, 200 CoCl_2_*6H_2_O, 20 CuCl_2_*2H_2_O, 20 NiCl_2_*6H_2_O, 40 Na_2_MoO_4_*2H_2_O. Rice straw hydrolysate was added to the sterilized medium to a final volume of 1 liter.

Cultivations of wild-type *A. baylyi* ADP1 or the *gcd* knockout strain of *A. baylyi* ADP1 were started by inoculating the media with precultivations to an OD_600_ of 0.02–0.06. The co-cultivations of the *gcd* knockout strain of *A. baylyi* ADP1 and *C. butyricum* were started by adding 0.235 ml of *A. baylyi* ADP1 *gcd* knockout precultivation and 0.370 ml of *C. butyricum* precultivation to initial OD_600_s of 0.2 and 0.5, respectively. Growth curves were determined spectrophotometrically with an Ultrospec 500 pro Visible spectrophotometer (GE Healthcare Bio-Sciences, Uppsala, Sweden) by measuring optical density at 600 nm. Sterile ion exchanged water was used in diluting the cultivations where necessary.

### Preparation of rice straw hydrolysate

Rice straw was used as lignocellulosic biomass and was subjected to mechanical and physical pretreatment. The rice straw was chopped and ground into very fine material and dry heated in a hot air oven at 120 °C for 2 h. It was then washed with large volumes of water until no measurable sugar was detected (using the dinitrosalicylic acid (DNS) assay [38]) in the wash, and then freeze dried. Commercial enzyme preparations in the form of Cellic^®^CTec2 and Cellic^®^HTec2 (Novozymes-cat. no’s VHN00003 and VCN10018) were used for the hydrolysis of the rice straw. The optimal enzyme ratio was determined by combining CTec2 (CT) and HTec2 (HT) in different (percentage) ratios (CT 100: HT 0), (CT 90: HT 10), (CT 70: HT 30), (CT 50: HT 50), (CT 0: HT 100). The total protein concentration was always maintained at 1 mg/ml in a total reaction volume of 10 ml and the final substrate concentration was kept at 2 % (w/v). The reaction was carried out at pH 5.0 using citrate buffer (100 mM) and the reaction mixture was incubated for 48 and 96 h at 50 °C and shaking at 150 rpm. The amount of reducing sugar produced was estimated using the dinitrosalicylic acid (DNS) assay [38]. Based on the optimization studies, the optimal mixture for the maximum release of reducing sugars from rice straw was (CT 90: HT 10), (after 96 h of incubation). The optimal enzyme ratios were validated by scaling up the total reaction volume to 100 ml. However, due to cost considerations, it was decided to conduct the large scale hydrolysis of rice straw (2 % w/v) using a combination of (CT 70: HT 30) at a protein concentration of 1 mg/ml. The final volume of the reaction mixture was 1 l and the reaction was carried out at pH 5.0 (Citrate buffer, 100 mM), 50 °C, and shaking at 150 rpm for 96 h. The residual biomass was separated by centrifugation at 12,000×*g* for 10 min at 4 °C. The hydrolysate was used for subsequent studies.

### Measurement of metabolite concentrations

High-performance liquid chromatography (HPLC) was used in determining the concentrations of d-glucose, d-xylose, l-arabinose, acetate, formate, and levulinate. The analysis was carried out as described in Santala et al. [25] but the column used was a Rezex™ RHM-Monosaccharide H+ (8 %) column (Phenomenex, Torrance, CA, USA). The column was used at 40 °C with 5 mM H_2_SO_4_ as an eluent (pumping rate 0.6 ml/min). The HPLC apparatus consisted of LC-20AC prominence liquid chromatograph (Shimadzu, USA), CBM-20A prominence communications bus module, SIL-20AC prominence autosampler, DGU-20A5 prominence degasser, and RID-10A refractive index detector. The HPLC samples were centrifuged for 5 min at 25,000 g and the supernatants were filtered (Chromafil^®^ PET -45/25, Macherey–Nagel, Germany) prior to analysis.

Gas chromatograph (GC-2014, Shimadzu GC) using Instrument N_2_ 5.0 as the carrier gas (flow rate, 20 ml/min), equipped with thermal conductivity detector (operating temperature, 110 °C) and PORAPAK column (length, 2 m; inner diameter, 2 mm; operating temperature, 80 °C) was used to determine the gaseous composition from fermentation experiments. The headspace H_2_ concentration was calculated by converting the H_2_ data (ml) obtained from GC to milli molar (mM) values using the ideal gas law equation (for room temperature) and volume of cultivation medium (l) [39].

The concentrations of 4-hydroxybenzoate were determined spectrophotometrically with a NanoDrop 2000 UV–Vis spectrophotometer (NanoDrop Products, Wilmington, DE, USA) by measuring the absorbance at 280 nm. The analysis is based on the fact that aromatic compounds absorb light at UV wavelengths and has been successfully used by Dal et al. [21] with *A. baylyi* ADP1. A standard curve (0.05, 0.1, 0.5, 1, 2, 5, 7.5, 10, 12.5, and 15 mM) was prepared in the medium used in the experiment where 4-hydroxybenzoate was studied and the standard curve was determined in triplicate. As a control, we measured absorbance at 280 nm during a cultivation lacking added 4-hydroxybenzoate and we did not obtain values above the lowest point of the standard curve.

## Abbreviations

HPLC: high-performance liquid chromatography
GC: gas chromatography
rpm: revolutions per minute
CT: CTec2
HT: HTec2
